# Glucagon-Like Peptide-1 Analog Liraglutide Protects against Diabetic Cardiomyopathy by the Inhibition of the Endoplasmic Reticulum Stress Pathway

**DOI:** 10.1155/2013/630537

**Published:** 2013-03-05

**Authors:** Jieyu Liu, Yu Liu, Li Chen, Yuehui Wang, Junqi Li

**Affiliations:** ^1^Department of the Endocrinology, The Second Hospital of Jilin University, Jilin University, Changchun 130041, China; ^2^Department of the Pharmacology, Norman Bethune Medical College, Jilin University, Changchun 130021, China; ^3^Department of the Cardiovascular, The Second Hospital of Jilin University, Jilin University, Changchun 130041, China

## Abstract

*Aim*. This study aimed to investigate whether the glucagon-like peptide-1 analog liraglutide (LIRA) can protect against diabetic cardiomyopathy and explore the related mechanism. *Methods*. Rats were divided into 6 groups: a nondiabetic group, diabetic cardiomyopathy rats without LIRA treatment, diabetic cardiomyopathy rats with LIRA treatment (with high-, medium-, and low-dose, resp.), and diabetic cardiomyopathy rats treated with insulin. Cardiac function was examined by echocardiography before and after treatment. The histopathology of the heart was examined with H&E staining. The mRNA levels of XBP1, ATF4, and TRAF2 were analyzed by RT-PCR, and the expression of glucose-regulated protein 78 (Grp78), enhancer-binding protein homologous protein (CHOP), caspase-3, and caspase-12 was detected by western blot. *Results*. LIRA strongly improved cardiac function from both echocardiographic and histopathologic analyses, but insulin only partly increased cardiac function by improving FS and LVPW values. LIRA treatment can significantly decrease the expression of XBP1, ATF4, and TRAF2 (*P* < 0.01). LIRA also significantly downregulates the expression of Grp78, caspase-3 (*P* < 0.01), CHOP, and caspase-12 (*P* < 0.05). *Conclusions*. LIRA can protect against diabetic cardiomyopathy by inactivating the ER stress pathway. The improvement in cardiac function by LIRA is independent of glucose control.

## 1. Introduction

Diabetes mellitus is a serious, complex metabolic disease that affects approximately 4% of the population worldwide [1]. Cardiovascular complications are the leading cause of diabetes-related morbidity and mortality [2]. Although coronary atherosclerosis is the major cause of cardiovascular diseases in diabetic patients, diabetic cardiomyopathy increases the risk of heart failure independently of coexisting coronary artery disease, obesity, and hypertension [3, 4]. Diabetic cardiomyopathy refers to a disease process which affects the myocardium in diabetic patients causing a wide range of structural abnormalities, such as ventricular dilation prominent interstitial fibrosis and cardiomyocyte hypertrophy [5, 6], and eventually leads to left ventricular hypertrophy and diastolic and systolic dysfunction or a combination of these [7, 8]. The prevalence of diabetic cardiomyopathy may reach to ~60% in type 2 diabetic patients [9].

The pathogenesis of diabetic cardiomyopathy was intensively investigated during the past decade. Impaired calcium homeostasis, myocardial insulin resistance, increased lipid uptake, glucotoxicity, activation of the renin-angiotensin system, and increased oxidative stress are the major mechanisms [10]. Furthermore, the pathological role of endoplasmic reticulum (ER) stress is increasingly recognized. 

The ER is a central organelle entrusted with lipid synthesis, protein folding, and protein maturation, and the ER is involved in the intrinsic pathway of apoptosis [11]. Various conditions, including hypoxia, ischemia, elevated protein synthesis, exposure to free radicals, hyperhomocysteinemia, and gene mutations, can induce the pathological accumulation of unfolded proteins in the ER, a condition referred to as ER stress [12, 13]. ER stress plays a role in many pathological conditions, such as tumors, viral diseases, prion disease [14], and diabetic kidney disease [15]. Some complex homeostatic signaling pathways, such as the unfolded protein response (UPR), have evolved to deal with ER stress [16]. One action of UPR is to activate the expression of glucose-regulated protein 78 (Grp78), which is an ER resident protein that plays an important role in dealing with accumulated proteins. Moderate ER stress could alleviate injury triggered by stress, but severe and chronic stress could lead to apoptosis and induce many diseases. The activation of JNK and the transcriptional induction of CHOP and caspase-12-dependent pathways could initiate apoptotic processes [17, 18]. Recently more studies have strongly demonstrated the critical role of ER stress in the development of diabetic cardiomyopathy. The experimental evidence has been provided that two ER stress hallmarks, GRP78, and caspase-12 were upregulated in the diabetic rat hearts compared to normal rat hearts [19]. Whereas some drugs such as valsartan could relieve the ER stress-associated apoptosis, resulting in a significant prevention of cardiac remodeling [20].

Glucagon-like peptide 1 (GLP-1), a major incretin hormone, is released from L cells in gut in response to nutrients and potently stimulates glucose-induced insulin secretion [21]. In patients with type 2 diabetes, its secretion is diminished [22, 23], and incretin-based therapies have emerged as an important therapeutic option. Activation of the GLP-1 receptors enhanced insulin synthesis/secretion, suppressed glucagon secretion, slowed gastric emptying, and enhanced satiety [24]. 

The GLP-1R is fairly widely expressed in heart and vasculature [25, 26]. Therefore, in addition to its incretin effect, studies in both animals and humans have repeatedly shown a beneficial action of GLP-1 on cardiovascular system. Recent evidence has confirmed that GLP-1 increases myocardial glucose uptake independently of its ability to enhance insulin secretion [27–29] and increase survival of cardiac cells and cardiac function in rat models [30]. Treatment with GLP-1 analog liraglutide (LIRA) for 2 weeks in db/db mice downregulated genes involved in proapoptosis and endoplasmic reticulum (ER) stress [31].

In the current study, we investigated whether the GLP-1 analog, LIRA, improves diabetic cardiomyopathy in the STZ- induced diabetic rats. Our results indicate that GLP-1 analog, LIRA, improves cardiac function via the inhibition of ER stress in the rats with diabetic cardiomyopathy. 

## 2. Materials and Methods

### 2.1. Animals Preparations and Experimental Protocol

Adult male SD rats (Animal Center of Jilin University, Changchun, Jilin Province), weighting 200–250 g, were studied. Rats were housed at 20–22°C on a 12-h light-dark cycle. Rats were separated into high-fat diet rats (*n* = 110) and control rats (*n* = 10). The former were fed with high-fat diet for 8 weeks and then given intraperitoneal injection of streptozotocin twice (STZ at 30 mg/kg, Sigma-Aldrich, USA, dissolved in citrate buffer, pH 4.5), and the latter were fed with regular chow and injected with the same dose of citrate buffer. Five weeks after the STZ injections, blood samples were harvested from the rat tail vein after 12 h of fasting. The levels of fasting blood glucose (FBG) were measured in spectrophotometry-based assays using commercially available kits (Invitrogen, USA). Those rats with FBG > 7.8 mmol/L were considered to be diabetic rats. The FBG of control rats is normal. STZ-induced diabetic rats were randomly studied in the following 5 different treated groups, non-LIRA group (DCM, *n* = 8), high dose of LIRA group (LH, 500 *μ*g/kg, *n* = 10), medium dose of LIRA group (LM, 100 *μ*g/kg, *n* = 12), low dose of LIRA group (LL, 50 *μ*g/kg, *n* = 10), and insulin group (glargine 3.2 IU/kg, *n* = 8). At 8 weeks after LIRA or insulin treatment, rats were euthanized with ketamine HCl (50 mg/kg) and xylazine (10 mg/kg) for study. The levels of FBG, body weight, and cardiac function were measured by echocardiography at baseline and 8 weeks of treatment, and heart tissues were collected after 8 weeks of treatment. The investigation conforms to the Guide for the Care and Management of Laboratory Animals published by the Universities Federation for Animal Welfare (UFAW). The study protocols were approved by the Animal Care and Use Committee of the University of Jilin.

### 2.2. Echocardiographic Evaluation

Rats were measured with echocardiography to compare the development of diabetic cardiomyopathy. Two-dimensional and M-mode echocardiography images of rats were obtained using a commercially available 12 MHz linear array transducer system and an echocardiogram machine (HP, USA). M-mode recordings were of the left ventricle (LV) at the level of the mitral valve in the parasternal view using two-dimensional echocardiography guidance in both the short- and long-axis views. Pulsed-wave Doppler was used to examine mitral diastolic inflow in the apical four-chamber view. For each measurement, the data were averaged from three consecutive cardiac cycles. All measurements were made from digital images captured at the time of the study by the use of inherent analysis software (Sonos 5500 software packages). 

### 2.3. Tissue Preparation and Hematoxylin-Eosin Staining

The LV was removed and sectioned into four slices along a plane parallel to the atrioventricular ring. The middle section was fixed in 4% buffered formalin, and 4 *μ*m paraffin-embedded sections were prepared for hematoxylin-eosin (HE) staining. The remaining portion of the heart sample was stored at −80°C for western blot or semiquantitative reverse transcription PCR (RT-PCR) assays.

### 2.4. Western Blot Analysis

After extraction of myocardial proteins, equal amounts of the protein preparations were separated by 15% SDS-PAGE, as described in [32]. The separated proteins were transferred to nitrocellulose membranes (Invitrogen, USA) for 50 min at 120 V. The membrane was blocked with 5% nonfat milk in PBST (phosphate buffered saline, pH 7.6, containing 0.05% tween-20) for 2 h at room temperature and then incubated with a primary antibody against Grp78 (1 : 1000, Santa Cruz, USA), caspase-3 (1 : 1000, Sigma, USA), CHOP (1 : 500 and 1 : 1000, resp., Stressgen, USA), and *β*-actin (1 : 600, Santa Cruz, USA) at 4°C overnight. After incubating with 1 : 4000 horseradish-peroxidase-(HRP-) conjugated anti-mouse/rabbit/goat IgG (Santa Cruz, USA), the blots were developed using enhanced chemiluminescence (PE Applied Biosystems, USA). The membranes were scanned densitometrically by Typhoon (Pharmacia, USA) and quantitated using Image Total Tech (Pharmacia, USA). 

### 2.5. RNA Extraction and Semiquantitative RT-PCR

To evaluate the transcription of the myocardial ER stress-related factors, such as XBP1, ATF4 and TRAF2, semi-quantitative RT-PCR assays were performed. Total RNA was extracted from frozen myocardial tissues using TRIzol (Invitrogen, Carlsbad, USA) according to the manufacturer's instructions. Primers specific for XBP1, ATF4, and TRAF2 were synthesized and listed below: XBP1: sense: TGGCCGGGTCTGCTGAGTCCG, antisense: ATCCATGGGAAGATGTTCTGG; ATF4: sense: GTTGGTCAGTGCCTCAGACA, antisense: CATTCGAAACAGAGCATCGA; TRAF2: sense: ACCTGTGATGGCTGTGGC, antisense: TCTGTGAGGCTTGGGACT. *β*-actin was used as the internal control. Total cellular RNA (including HepG2 and HL-7702) was prepared using an RNA simple Total RNA kit (TIANGEN, China). Reverse transcription was performed using the SuperScript III First-Strand Synthesis System (Invitrogen, USA), according to the manufacturer's protocol. Two microliters of RT reaction products was amplified by PCR in a volume of 50 *μ*L under the following conditions: 94°C for 40 s, 60°C for 30 s, and 72°C for 30 s. After electrophoresis on a 1.5% agarose gel, the gel images of each PCR product were digitally captured with a CCD camera and analyzed with the NIH Imager beta version 2. Relative transcriptional values for each factor in the semi-quantitative RT-PCR are presented as the ratio of the signal value of the specific PCR product to that of *β*-actin.

### 2.6. Statistical Analysis

All data are presented as means ± SD. Statistical analyses were performed with SPSS13.0 software, using either student *t*-test or analysis of variance (ANOVA) with post hoc analysis as appropriate. A *P* value <0.05 was considered statistically significant.

## 3. Results

### 3.1. Development of DCM

**Figure 1:**
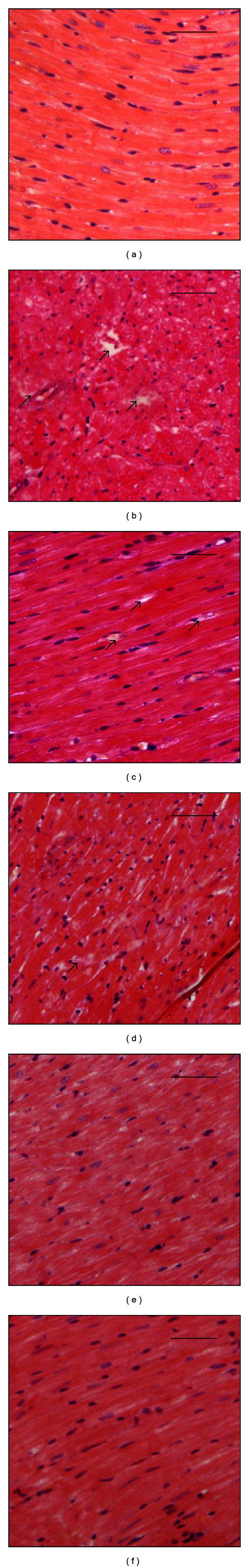
Histopathological improvement of LIRA-treated diabetic myocardium. The normal myocardium, diabetic myocardium and LIRA or ISN treated DCM myocardium were stained with HE staining (200x magnification). Representative samples of normal rats (a), diabetic cardiomyopathy rats (b), INS treated DCM rats (c), and LIRA treated DCM rats ((d) to (f)) were shown in the figure. Arrows indicate regions with ischemic myocyte degeneration in the subendocardial, subepicardial region and papillary muscles of the myocardium. Bar = 50 *μ*m.

The LV systolic parameters, including LV end-diastolic diameter (LVEDD) and LV posterior wall (LVPW), fractional shortening (FS), and ejection fraction (EF), significantly decreased in DCM group compared with CON group (Table 1). LV diastolic function variables expressed by the E-wave (early diastolic filling and early peak velocity) differed significantly in DCM rats compared with CON rats. A significant reduction in the E-wave velocity, a significant increase in the A-wave velocity, and a significant decrease in the E/A ratio was found, with an obvious decrease in FS and EF (Table 1). The altered cardiac diastolic performance is thought to result from reduced cardiac compliance. The results show that the diabetic cardiac muscle fibers were disordered, and many of the fibers were collapsed, according to the HE staining (Figures 1(a) and 1(b)). The levels of FBG in DCM rats were measured every week and showed a persistence of hyperglycemia of approximately 10 mmol/L from the 1st week after 5 weeks of two STZ treatments (hyperglycemic establishment) to the 8th week after hyperglycemia. Combined with the FBG data, histomorphological examination by HE staining confirmed the development of DCM.

### 3.2. LIRA Improves Cardiac Function of DCM Rats

As shown in Table 1, LVPW and LVEDD in DCM rats were significantly increased compared with CON rats (*P* < 0.05). However, upon long-term treatment with LIRA, LVPW and LVEDD in LIRA treated rats were significantly decreased (LL, *P* < 0.05 for both; LM, *P* < 0.05 for LVPW and *P* < 0.01 for LVEDD; LH, *P* < 0.01 for LVPW and *P* < 0.05 for LVEDD), compared with DCM rats. On the contrary, insulin (INS) decreased the LVPW level (Table 1, *P* < 0.01) but not the LVEDD level. EF and FS were improved significantly in the group of the long-term treatment with middle dose of LIRA (*P* < 0.05 for EF, *P* < 0.01 for FS, resp.). INS also improved the FS reduction in DM rats (*P* < 0.01) without the effect on EF. In the four treated groups (INS, LL, LM, and LH), only LM group had a marked improvement in the E/A ratio (*P* < 0.01). There was no change in the FBG level between DCM with and without the 8-week LIRA treatment. According to the HE staining results from the cardiac myocytes, we found that the disordered diabetic cardiac muscle fibers were repaired by the addition of LIRA, especially in the LM group and LH group (Figures 1(e) and 1(f)). The treatment with either INS or a low dose of LIRA could not strongly improve the cardiac structure (Figures 1(c) and 1(d)).

### 3.3. LIRA Inhibits Cardiac Myocyte Apoptosis in DCM Rats

**Figure 2:**
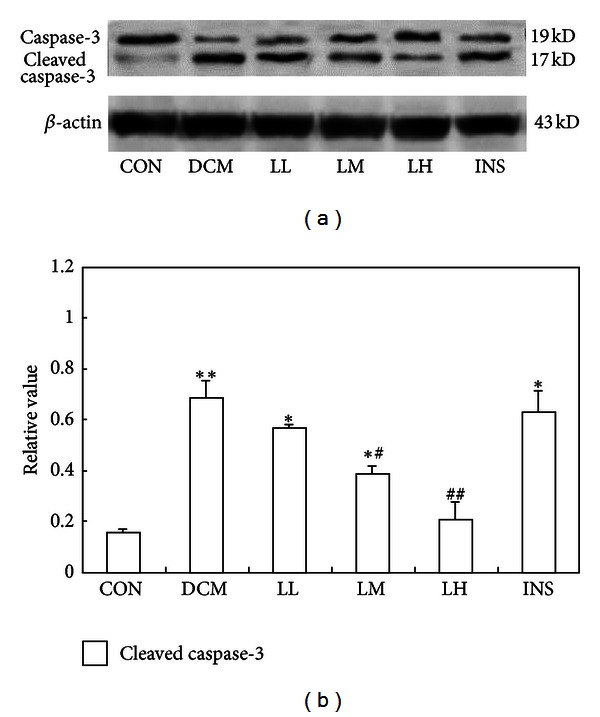
Effects of LIRA on the activation of caspase-3 in the DCM and CON. (a) Western blot was performed using each relevant antibody. *β*-actin was shown as a loading control. (b) Statistical analysis. Data were shown as mean ± S.D. *P < 0.05 and **P < 0.01 versus CON rats, ^#^ *P* < 0.05, and ^##^ *P* < 0.01 versus DCM rats.

The expression of Caspase-3 is measured to evaluate the apoptosis of cardiac myocyte since ER stress-associated apoptosis is significantly associated with cardiac remodeling. Our Western blotting showed that caspase-3 was activated significantly in DCM rats (Figure 2, *P* < 0.05). However, caspase-3 was significantly inhibited in LIRA-treated group, especially in the LM group, which shows Lira can decrease the apoptosis of cardiomyocyte. 

### 3.4. LIRA Decreases ER Stress-Induced Myocardial Apoptosis by Downregulating the Expression of CHOP and Grp78 and Inactivating Caspase-12

**Figure 3:**
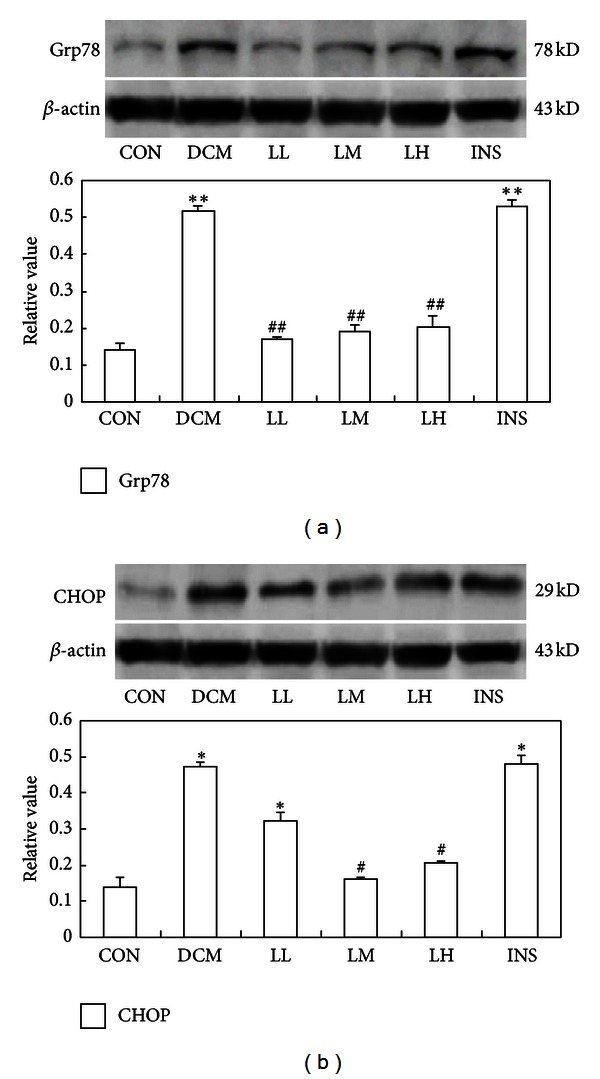
Immunoblot analysis for (a) Grp78 and (b) CHOP in the myocardium of DCM and CON rats. The upper trace of each panel shows representative blots of proteins in DCM and CON rats. The lower panels show the bar graphs summarizing the immunoblot data. Western blot was performed using each relevant antibody. *β*-actin was shown as a loading control. Data were shown as mean ± S.D. *P < 0.05 and **P < 0.01 versus CON rats, ^#^ *P* < 0.05, and ^##^ *P* < 0.01 versus DCM rats.

Grp78 is an important molecular chaperone localized in the ER, which is usually regarded as an indicator reflecting the activation of ER stress. In the DCM rats, Grp78 expression was abundant in myocardium, suggesting that myocardial ER stress is present in DCM rats. However, the Grp78 expression in DCM rats treated with LIRA was significantly attenuated compared with the DCM group (Figure 3(a), *P* < 0.05). Since CHOP is also essential for ER stress-induced cardiomyocyte apoptosis in diabetes, we next examined the expression of CHOP and shows that the expression of CHOP was in parallel with the incidence of cardiomyocyte apoptosis in DCM rats. Interestingly, LIRA treatment at the middle and high doses decreased the expression of CHOP (Figure 3(b), LM and LH groups, both *P* < 0.05). Low-dose LIRA and insulin had no effect on CHOP expression (Figure 3(b)).

**Figure 4:**
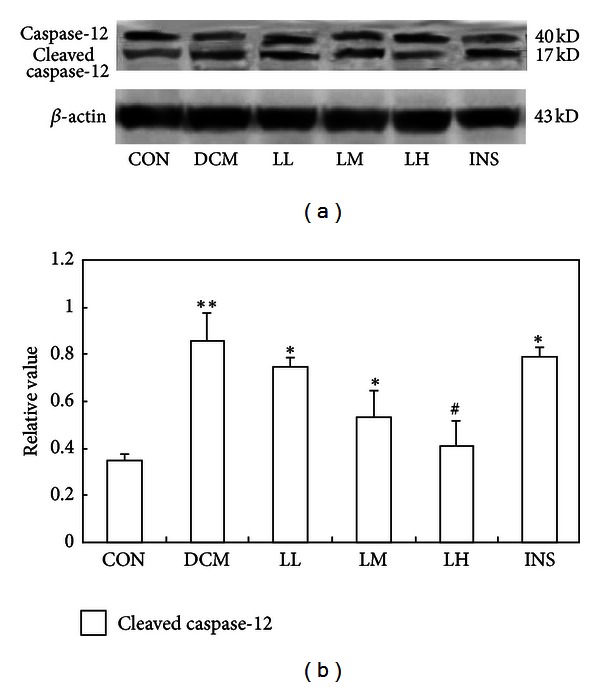
Effects of LIRA on the activation of caspase-12 in the DCM and CON. (a) Western blot was performed using each relevant antibody. *β*-actin was shown as a loading control. (b) Statistical analysis. Data were shown as mean ± S.D. *P < 0.05 and **P < 0.01 versus CON rats, ^#^ *P* < 0.05 versus DCM rats.

To further certify the ER stress, we observed caspase-12 activation in DCM rats. The results showed that caspase-12 activity was significantly increased in DCM rats compared with the CON (Figure 4, *P* < 0.01). When DCM rats were treated, only the LH group, but not the other treatment groups (including the LL, LM, and INS groups), showed a reduced level of caspase-12 activation (Figure 4, *P* < 0.05). 

### 3.5. LIRA Blocks the mRNA Transcription of Some ER Stress-Associated Factors

**Figure 5:**
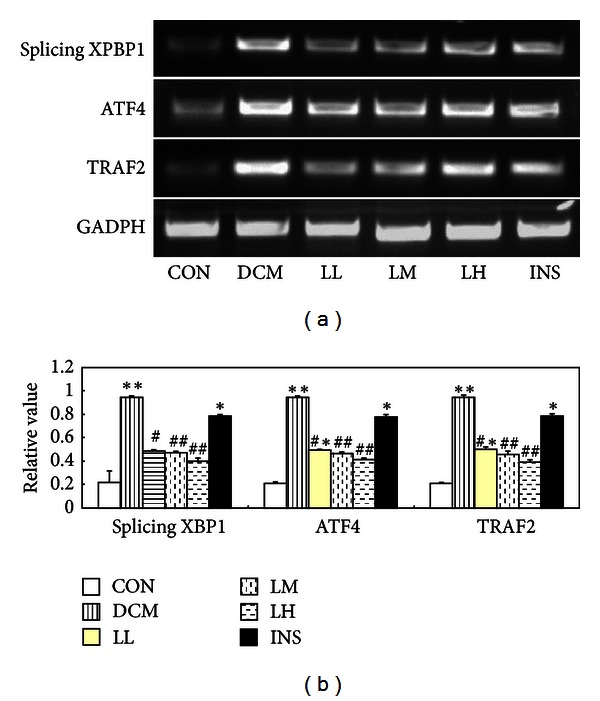
Semiquantification of mRNA levels of the ER stress-associated genes in the DCM and CON. (a) The ATF4, TRAF2, and splicing XBP1 were separated in 1.4% agarose gels. The relative value of each preparation is calculated by the gray numerical value of each specific product versus that of *β*-actin. (b) The average data of each preparation are evaluated based on three independent reactions and represented as mean ± S.D. Statistical differences of the data of each preparation compared with that of CON are illustrated as *P < 0.05 and **P < 0.01, and of DCM are illustrated as ^#^ *P* < 0.05 and ^##^ *P* < 0.01, respectively.

Because those factors of ATF4, TRAF2, and XBP1 are related to ER stress, we further analyzed their transcriptional levels using semi-quantitative RT-PCR. As shown in Figure 5, mRNA levels of ATF4, TRAF2, and XBP1 were significantly higher in the heart of DCM rats than that in CON rats (*P* < 0.05). On the contrary, LIRA treatment decreased them. INS did not change the levels. 

## 4. Discussion

The current study demonstrates that GLP-1 exerts cardioprotective actions in experimental models of diabetic cardiomyopathy. While diabetes leads to myocardial structural and function abnormalities in vivo, administration of GLP-1 analog, LIRA, improved the disorder of cardiac muscle fibers and LV diastolic and systolic parameters. LIRA decreased some mRNA expression of ER stress-related factors and ER stress, which are associated with myocardial apoptosis. Thus, our data strongly suggest that activation of GLP-1 receptor protects against diabetic cardiomyopathy by the inhibition of the endoplasmic reticulum stress pathway. 

Prior studies have demonstrated the presence of the GLP-1 receptor in the myocardium and even an increase in GLP-1 receptor in DCM [33]. This supports the idea that GLP-1 contributed to the beneficial extrapancreatic effects on heart independently of its role in pancreatic insulin release. The current study further extends previous findings by demonstrating that activation of the GLP-1 receptor is beneficial to diabetic cardiomyopathy through decreasing ER stress. 

Recent studies indicated that hyperglycemia-caused ER stress played an important role in diabetic cardiomyopathy [34], which is consistent with our study. The ER plays an essential role in the modification process after protein synthesis and is also where the disposal of abnormally folded proteins begins. Normally, the unfolded protein response (UPR) could result in upregulation of ER stress-associated chaperone synthesis. In the diabetic cardiomyopathy rats, according to the metabolism drawback, the proteins in cardiac myocytes may suffer from UPR and trigger an increase in Grp78 protein. Actually, in our study, Grp78 is involved in ER stress in DCM rats and LIRA is responsible for the inhibition of Grp78 expression. Grp78 protein is an important molecular chaperone localized in the ER, which refers to the immunoglobulin heavy chain binding protein (Bip) and plays a vital role in the recognition of unfolded proteins. Grp78 also serves as a master modulator for the UPR network by binding to ER sensors, such as protein-kinase-R-(PKR-) like ER kinase, inositol-requiring 1 (IRE1). Activation of IRE1 induces X-box-binding protein mRNA splicing. The spliced XBP1 protein functions as a transcription factor, which induces the ER stress gene Grp78. We detected XBP1 mRNA, which is also overexpressed in the DCM rats and inactivated in the LIRA-treated DCM rats. This result is consistent with the upregulation of Grp78.

CHOP is the downstream protein of the apoptotic pathway and plays an important role in ER stress-induced apoptosis. CHOP can be activated by the overtranscription of ATF4, TRAF2, and XBP1 [35, 36]. Accumulation of CHOP can promote the transcription of ATF4, TRAF2, and XBP1, and overexpression of this factor can sensitize the ER stress of cells via increasing the expression of the CHOP protein. To explore the antidiabetic cardiomyopathy function of LIRA in DCM, CHOP is a key target. Our results showed that the expression of CHOP was significantly upregulated in DCM rats compared with CON rats. The overexpression of CHOP in DCM rats can be significantly inhibited by a medium and high dose of liraglutide (LM and LH groups), but there are no obvious changes in the LL group, which shows a dose effect of LIRA on the inhibition of the CHOP expression 

Caspase-12 is exclusively located at the ER, and, following its activation, it can directly process downstream caspases in the cytosol, mainly caspase-9 and caspase-3 [37]. Caspase-12 mediated apoptosis is a specific apoptosis pathway of the ER, and apoptosis that occurred because of membrane or mitochondrial targeted signals would not activate caspase-12 [38]. In this study, caspase-12 also participates in ER stress in the DCM rats through enhancing its activity. This activated caspase-12 could only be blocked significantly by the addition of high-dose LIRA (LH group), which showed the same dose-dependent effect as CHOP protein. The release of TRAF2 is believed to be a biomarker for ER stress, which activates caspase-12. Furthermore, TRAF2-JNK is the third pathway in ER-associated apoptosis, which has been demonstrated to be vital in insulin resistance. Perhaps the upregulated mRNA of TRAF2 induced the activation of caspase-12 and the JNK apoptotic pathway. LIRA could inhibit ER stress via downregulating TRAF2 directly or indirectly. In our study, the increased induction of Grp78 and CHOP, the cleavage of caspase-12, and JNK phosphorylation in diabetic cardiomyopathic rats paralleled with the destruction of cardiac function. The mechanism of the protection in LIRA group was triggered by the inactivation of the ER stress pathway, including CHOP, caspase-12, and JNK pathway in diabetic cardiomyopathic rats. However, the improvements in cardiac function by LIRA are independent of glucose control.

We employed echocardiography to evaluate the influence of LIRA on cardiac function for the first time and creatively found that the treatment of LIRA could protect against diabetic cardiomyopathy. It is interesting to note that, although high-dose LIRA showed the strongest effect on the inhibition of ER stress and histopathologic analysis also supported the results that high-dose LIRA could improve cardiac structure significantly, the echocardiographic evaluation showed that high-dose LIRA only decreased the parameters of LVPW and LVEDD. In contrast, insulin did not have significant effect on cardiac structure by histopathologic analysis, which was consistent with the results of ER stress. However, from echocardiography detection, insulin could improve the values of FS and LVPW, indicating a minor improvement on cardiac functions. All of this implied that the echocardiography results are not always paralleled with the molecular and histopathologic analyses. The phenomenon is worthy to be studied in detail in the future.

## 5. Conclusions

In conclusion, GLP-1 improves diabetic cardiomyopathy and heart function by the decrease of cardiac myocyte ER stress and subsequent myocardial apoptosis. This is an important finding because ER stress is one of the underlying mechanisms of diabetic complications. Thus, chronic treatment of GLP-1 analogs may significantly contribute to complication prevention in diabetes.

## Figures and Tables

**Table 1:** Echocardiographic and fasting blood glucose data.

	CON	DCM	INS	LL	LM	LH
*n*	4	4	4	7	8	8
FBG (mmol/L)	2.83 ± 0.39	10.39 ± 1.78^b^	8.49 ± 1.86^a^	8.6 ± 3.7^a^	7.8 ± 1.86^a^	7.47 ± 2.95^a^
E/A	2.45 ± 0.99	0.32 ± 0.04^a^	0.39 ± 0.06^a^	0.31 ± 0.03^a^	1.38 ± 0.09^ad^	0.35 ± 0.1^a^
EF	88.25 ± 0.96	75.75 ± 4.57^b^	88.5 ± 0.58	74.56 ± 6.96^a^	87.63 ± 5.97^c^	75.13 ± 2.95^b^
FS	52.25 ± 0.96	44.5 ± 2.52^b^	53.25 ± 0.5^d^	39.33 ± 8.46^b^	52.13 ± 3.76^d^	38.88 ± 2.3^b^
LVPW (mm)	3.47 ± 0.4	4.53 ± 0.5^a^	3.23 ± 0.17^d^	3.31 ± 0.38^c^	3.3 ± 0.42^c^	2.72 ± 0.31^ad^
LVEDD (mm)	5.54 ± 0.99	6.54 ± 0.35^a^	6.27 ± 0.68^a^	5.47 ± 0.59^c^	4.88 ± 0.76^ad^	5.13 ± 0.26^c^

LVEDD: left ventricular end-diastolic diameter; LVPW: left ventricular posterior wall; E/A: the ratio of E and A (E: peak early transmitral filling velocity during early diastole; A: peak transmitral atrial filling velocity during late diastole); FS: fractional shortening; EF: ejection fraction; FBG: fasting blood glucose. ^a^ *P* < 0.05 versus CON rats; ^b^ *P* < 0.01 versus CON rats; ^c^ *P* < 0.05 versus DCM rats; ^d^ *P* < 0.01 versus DCM rats.

## Abbreviations

CHOP: Enhancer-binding protein homologous protein
CON: Nondiabetic cardiomyopathy
DCM: Diabetic cardiomyopathy
E/A: The ratio of E and A
EF: Ejection fraction
ER: Endoplasmic reticulum
FS: Factional shortening
GLP-1: Glucagon like peptide-1
Grp78: Glucose-regulated protein 78
INS: Diabetic cardiomyopathy rats with insulin treatment
IRE1: Inositol-requiring 1
LH: Diabetic cardiomyopathy rats with LIRA high-dose treatment
LIRA: Liraglutide
LL: Diabetic cardiomyopathy rats with LIRA low-dose treatment
LM: Diabetic cardiomyopathy rats with LIRA medium-dose treatment
LVEDD: Left ventricular end-diastolic diameter
LVPW: Left ventricular posterior wall
PKR: Protein-kinase-R
UPR: Unfolded protein response.
